# Participation in a Fruit and Vegetable Prescription Program for Pediatric Patients is Positively Associated with Farmers’ Market Shopping

**DOI:** 10.3390/ijerph17124202

**Published:** 2020-06-12

**Authors:** Amy Saxe-Custack, Richard Sadler, Jenny LaChance, Mona Hanna-Attisha, Tiffany Ceja

**Affiliations:** 1 Department of Food Science and Human Nutrition, Division of Public Health, Michigan State University-Hurley Children’s Hospital Pediatric Public Health Initiative, Flint, MI 48502, USA; 2 Department of Family Medicine, Division of Public Health, Michigan State University College of Human Medicine, Flint, MI 48502, USA; sadlerr@msu.edu; 3 Division of Public Health, Michigan State University-Hurley Children’s Hospital Pediatric Public Health Initiative, Flint, MI 48502, USA; jlachan1@hurleymc.com (J.L.); cejatiff@msu.edu (T.C.); 4 Department of Pediatrics and Human Development, Division of Public Health, Michigan State University-Hurley Children’s Hospital Pediatric Public Health Initiative, Flint, MI 48502, USA; hannamon@msu.edu

**Keywords:** nutrition, children, fruits and vegetables, farmers’ market

## Abstract

*Objectives*: The primary objective was to investigate the association between participation in a farmers’ market fruit and vegetable prescription program (FVPP) for pediatric patients and farmers’ market shopping. *Methods*: This survey-based cross-sectional study assessed data from a convenience sample of 157 caregivers at an urban pediatric clinic co-located with a farmers’ market. Prescription redemption was restricted to the farmers’ market. Data were examined using chi-square analysis and independent samples t-tests. *Results*: Approximately 65% of respondents participated in the FVPP. Those who received one or more prescriptions were significantly more likely to shop at the farmers’ market during the previous month when compared to those who never received a prescription (*p* = 0.005). *Conclusions*: This is the first study to demonstrate that participation in a FVPP for pediatric patients is positively associated with farmers’ market shopping.

## 1. Introduction

Diets rich in fruits and vegetables are necessary to support healthy growth and development [1,2,3], and prevent chronic disease [4,5,6]. Despite this, intake among USA children, particularly those from low-income households, fails to meet recommendations [7,8]. With childhood consistently identified as a critical period for the establishment of lifelong dietary patterns [8,9,10], public health efforts should address barriers to fruit and vegetable consumption among youth. Although knowledge deficits are certainly a concern [11], general nutrition education cannot be the sole consideration since many children face persistent struggles with food access and affordability [12,13]. To directly address these challenges, some health care practices have implemented farmers’ market fruit and vegetable prescription programs (FVPPs) [12,14]. Much like traditional prescriptions, physicians write the prescription, which is then exchanged for fresh produce at a local farmers’ market.

Farmers’ market shopping is directly related to the purchase and consumption of fruits and vegetables [15,16]. Therefore, programs that successfully draw children and families to local farmers’ markets have the potential to positively influence dietary intake. Unlike food shopping with children at convenience and grocery stores—which can induce requests for nutrient-poor snack foods [17]—shopping at a farmers’ market with a fruit and vegetable prescription intentionally directs children to fresh, high-nutrient foods. Although farmers’ market monetary incentive programs for adults are associated with increased purchasing of fresh produce from local markets [18,19,20,21], it is unclear whether farmers’ market FVPPs for children have the same effect.

Previous research related to FVPPs has primarily focused on programs that target income-eligible adults with diet-related health conditions, such as diabetes or heart disease [14,22]. Although few studies have examined FVPPs directed at children, early results suggest that exposure to pediatric FVPPs is associated with improvements in perceived and measured household food security [12,23], access to fresh foods [12,23], and child dietary patterns [12,24,25]. The current study is the first to investigate the relationship between participation in a farmers’ market FVPP for pediatric patients and farmers’ market shopping.

## 2. Materials and Methods

### 2.1. Study Population and Design

Nearly 60% of children who reside in Flint, Michigan live in poverty [26] and the community has a limited number of full-service grocery stores operating within the city [27]. In August 2015, the Hurley Children’s Center (HCC), a Michigan State University-affiliated residency training pediatric clinic with more than 11,000 visits per year, relocated to the downtown Flint Farmers’ Market (FFM), a move that increased the percentage of people coming by bus from the city’s poorest neighborhoods for general groceries [28]. The FFM is a year-round market with over 50 vendors who sell products inside and outside of the market building. Most vendors are local farmers who sell fresh produce, but the FFM also offers a meat and poultry market, breads and baked goods, cheeses, and several food stands. The co-location of one of the largest pediatric clinics in Flint with the downtown farmers’ market was an intentional effort to actively address persistent challenges with child access to fresh, high-quality foods. The HCC’s patient population is approximately half female (51%), majority (73%) are African American, and over 85% have Medicaid as their insurance.

Shortly after the relocation, the HCC partnered with the FFM to implement a FVPP for pediatric patients [12]. The program included one $10 prescription that may be redeemed only for fresh fruits and vegetables at the FFM. When the $10 prescriptions were introduced at the HCC in May 2016, eligibility was limited to well-child visits. Approximately one month later, the FVPP was expanded to include both well- and sick-child visits to effectively increase the number of children served by the program. One prescription for fruits and vegetables was then provided to every child at each office visit. Because the FVPP was provided only during well-child visits when it was introduced at HCC, some pediatric patients had not received prescriptions prior to enrollment in the current study.

This cross-sectional study enrolled a convenience sample of 157 caregivers of children presenting for care at the HCC. To be eligible for inclusion, participants had to be 18 years of age or older, English-speaking, and have one or more children who were active patients at the HCC. Trained clinic staff recruited participants from the HCC waiting room between June and August 2017, approximately one year after the implementation of the prescription program.

### 2.2. Data and Instrumentation

After reviewing the implied consent letter, study participants completed a 42-item survey. The survey took approximately 30 min to complete, and trained clinic staff were available to assist with survey completion. Survey items included questions from previously validated instruments related to food security and food access as well as questions related to caregiver and child characteristics, participation in food assistance programs, participation in the prescription program, and farmers’ market shopping. Caregivers were also asked to report their address or nearest intersection, from which we defined residence in Flint or not.

Household participation in the FVPP was measured with a single question that asked caregivers whether any of their children had received a fruit and vegetable prescription from the HCC. The primary outcome of interest was farmers’ market shopping during the previous month. The survey question asked, “Have you ever shopped at the Flint Farmers’ Market before?”, and the answer choices were “Yes, in past week”, “Yes, in past month”, “Yes, in past year”, “Yes, over a year ago”, and “Never”. Binary indicators were created to specify farmers’ market shopping within the previous month and year. The USA. Household Food Security Module: Six Item Short Form was used to measure financially-based food insecurity and hunger [29]. The sum of affirmative responses served as the household’s raw score. Food security status was assigned based on this calculated raw score (0–1 = high/marginal food security; 2–4 = low food security; 5–6 = very low food security). To evaluate specific access to fruits and vegetables, caregivers completed four questions from the Michigan Behavioral Risk Factor Surveillance Survey (MBRFSS) related to fruit and vegetable quality and access in neighborhood stores. Responses were answered on a 5-point Likert scale (1 = “always” to 5 = “never”).

Because evidence suggests that the neighborhood food environment (NFE) influences dietary habits [30,31,32], this relationship was also considered among our sample. In a previous study of Flint’s NFE, a modified Nutrition Environment Measures Survey in Stores was deployed at every store in and around the city. Each store was scored, representing a composite of the availability, quality, and variety of healthy foods (including versus less healthy options) in the store. These scores were linked to the geocoded site of each store, and a kernel density analysis was run to generate a continuous, interpolated surface. Effectively, areas with a greater density of stores having better availability, quality, and affordability of healthy foods had higher NFE scores. The minimum NFE score possible was 0 and the maximum NFE score possible was 1270. In the current study, we geocoded the home location of every pediatric patient involved in the FVPP and extracted the NFE score present at that point [33].

### 2.3. Statistical Analysis

Within the study time frame, we estimated that there would be 700 caregivers who brought children to appointments. We calculated that a sample of at least 124 caregivers would be needed to have a 95% confidence level with a margin of error of 8% to estimate our outcome of shopping at the FFM. Frequencies and percentages were calculated from demographic data to describe characteristics of caregivers who completed the survey. When examining differences between prescription program participants and non-participants, subjects were excluded if data were missing for any variable involved in the analysis. Analyses, including NFE, were conducted only for our records with a home address or street intersection within Flint. Analyses included chi-square, independent samples t-tests, and logistic regression using Statistical Package for the Social Sciences (version 24, IBM Corp., Armonk, NY, USA, 2016) with significance set at *p* < 0.05. Researchers received approval for the study from Hurley Medical Center Institutional Review Board (1070530-1). The study was carried out in accordance with the Ethical Principles established by the Declaration of Helsinki.

## 3. Results

Surveys were collected from 157 caregivers of 278 pediatric patients who ranged from 0 to 19 years of age. The mean number of children per caregiver was 2.35 ± 1.03. The majority of respondents were female (93%) and residents of Flint (74%), with approximately half (48%) reporting a high school education or less (Table 1). Most survey respondents (63%) were receiving benefits from the Supplemental Nutrition Assistance Program (SNAP). For respondents who reported receiving SNAP, 30% did not receive benefits from the Special Supplemental Nutrition Program for Women, Infants and Children (WIC) or Double-Up Food Bucks (DUFB), 20% received both WIC and DUFB, 15% received DUFB and not WIC, and 35% received WIC and not DUFB.

### 3.1. Participation in the Pediatric FVPP

Table 2 describes differences in caregiver and child characteristics based on participation in the FVPP. There were statistically significant differences (*p* < 0.05) between participants and non-participants with regard to caregiver gender, city of residence, and child race.

### 3.2. Farmers’ Market Shopping

Approximately 65% of caregivers who completed the survey indicated that their child had received at least one fruit and vegetable prescription at the HCC. Participants were significantly more likely than non-participants to receive benefits through WIC (*p* < 0.001), but differences in SNAP participation were not significant.

As shown in Table 3, caregivers who reported that their child had received a fruit and vegetable prescription were significantly more likely to report shopping at the farmers’ market during the previous month when compared to caregivers whose child had never received a prescription (50.6% versus 26.8%, respectively; *p* = 0.005). Similarly, caregivers who reported that their child had received a fruit and vegetable prescription were significantly more likely to report shopping at the farmers’ market during the previous year when compared with caregivers who reported that their child had never received a prescription (75.3% versus 53.6%, respectively; *p* = 0.007). A logistic regression analysis was done to examine what influences having shopped at the FFM in the last month; statistically significant characteristics (WIC participation, city of residence, caregiver gender, and child race) and having received at least one fruit and vegetable prescription were included as co-variates. The overall model fit the data (Hosmer-Lemoshow Goodness-of-fit statistic *p* = 0.965) and only having received at least one fruit and vegetable prescription was statistically significant (*p* = 0.003) when controlling for WIC (*p* = 0.817), city of residence (*p* = 0.740), caregiver gender (*p* = 0.374), and child race using the variables of African-American (*p* = 0.164), and Caucasian (*p* = 0.293).

### 3.3. Food Security

Nearly half of all caregivers (45%) who completed the survey indicated low or very low levels of household food security. As shown in Table 3, food security scores among caregivers who reported that their child had received a prescription (1.89 ± 2.06) were not significantly different from those who reported that their child had not received a prescription (1.75 ± 1.89).

### 3.4. Neighborhood Food Environment

The above characteristics were also cross-referenced with the neighborhood food environment (NFE). These scores were available only within the city limits of Flint, thus 102 families who had shared whether or not they had received a prescription met the criteria. Of the families included in the analysis, the average NFE score was 257 ± 238. Examining differences in NFE scores by participation in the program and redemption of prescriptions, there was no statistically significant difference in NFE scores with either, indicating that families in neighborhoods with poor food environment scores had no difference in use of the prescription program and the farmers’ market as compared to families living in neighborhoods with better food environment scores. Please see Table 3. Additionally, there was no difference in NFE score by food security groups.

## 4. Discussion

The current study is the first to demonstrate a positive association between child participation in a farmers’ market FVPP and farmers’ market shopping. This relationship remained consistent when controlling for potential confounding variables, such as participation in WIC, caregiver gender, city of residence, and child race. Findings support previous evidence that monetary incentives for fresh produce from local farmers’ markets are effective in increasing purchase and consumption of fresh fruits and vegetables [18,19,20,21]. Although seasonality of fruits and vegetables is also a determinant of intake [34], results of the current study indicate a significant association between participation in the year-round FVPP and farmers’ market shopping in the past year. Interestingly, this suggests that seasonality of fresh produce likely did not influence participation in the FVPP. Farmers’ markets, which provide easy access to fresh, high-quality foods [12,13,35], are a particularly important resource for minority children living in low-income households who are at an elevated risk for poor dietary behaviors [7,8,36]. In addition to providing early exposure to a wide variety of healthy foods, many farmers’ markets also support exposure activities for children, such as cooking classes and food tastings, which show strong potential to improve diet quality [37,38]. Farmers’ market-based nutrition education programs that focus on children have, in fact, been successful in increasing consumption of fruits and vegetables among participants [39]. Improved year-round access to fresh, high-nutrient foods as well as positive food experiences are notable benefits of pediatric FVPPs that necessitate a visit to a local farmers’ market.

Evidence suggests that higher fruit and vegetable consumption during childhood is associated with reductions in chronic diseases during adulthood [5,40], emphasizing the particular importance of programs that target children. Primary care physicians—who follow children from infancy to young adulthood—are well positioned to address food access and affordability challenges. This is crucial during childhood when dietary behaviors are established [9,10]. Uniquely different from current programs that focus largely on fruit and vegetable prescriptions as a disease-management approach for adults with diet-related chronic health conditions [14,22], the current study emphasized the critical role of fruits and vegetables in the prevention of chronic disease during formative childhood years [9,10,41]. This approach goes beyond traditional nutrition education to address persistent environmental challenges related to access and affordability of fresh produce.

Previous literature has demonstrated important differences between families of low socioeconomic status (SES) and those of higher SES when addressing home food environment [42,43,44,45,46]. For example, research has shown that children of mothers at the lowest educational levels ate fewer fruits and vegetables when compared with children of mothers at the highest educational levels [44]. Furthermore, research has demonstrated that mealtime structures, including families eating together, television viewing while eating, and sources of meals (restaurants, schools, home), are important in relation to child eating patterns and that caregivers influence child eating behaviors through their own behaviors, attitudes, and feeding styles [47]. Although the current study did not specifically assess dietary patterns in relation the participation in the FVPP, previous evidence indicated that the current program was perceived as effective in improving dietary patterns of participating children [12]. Future research will examine measured changes in dietary behaviors of caregivers and children in relation to their exposure to the FVPP.

With nearly half of caregivers in the current study reporting household food insecurity, results raise concerns about poor dietary patterns and food insecurity issues facing children in Flint. Furthermore, previous research in Flint has pointed to poor quality of produce available to residents who often struggle with additional challenges, such as limited transportation, that further compound access and affordability issues [12,13,48]. Because of these interconnections, we cross-referenced our data with NFE scores from previous work in Flint [33]. NFE scores were not significantly associated with program participation measures in our study, indicating that the quality of the food environment in one’s home neighborhood was not a significant predictor of participation (that is, people participated regardless of the context of their neighborhood food environment). This is additionally noteworthy because the HCC is co-located with the FFM, providing easy access to the farmers’ market after pediatric office visits. Future research will investigate this relationship among patients and families at a pediatric clinic that is located away from the downtown area and outside of the local farmers’ market.

Evidence suggests that fruit and vegetable intake is consistently and positively associated with income [8,41]; therefore, pediatric FVPPs are likely to disproportionately benefit low-income children and adolescents. Previous research in Flint has indicated that poor dietary patterns and food insecurity are pervasive issues among children living in this low-income, urban city [49]. Although the current study did not demonstrate a significant difference in food security scores between caregivers who reported that their child had received a prescription and those who did not, it is important to note that previous research has suggested that pediatric fruit and vegetable prescriptions may be an effective tool to improve dietary habits [12,25] as well as food security among low-income households [12,23]. Previous research demonstrating positive impacts of pediatric fruit and vegetable prescriptions on household food security differed from the current study in that eligibility was limited to children who were obese or overweight with distribution amounts based on household size [23]. Future research in Flint will investigate various FVPP models as well as caregiver– and child–reported changes in food security scores over time in relation to participation in prescription programs.

We acknowledge study limitations, including the lack of randomization, self-reported data, and small sample size. Additionally, selection bias may exist, although our analysis showed the characteristics of the study population closely matched those of the source patient population at the HCC which consists primarily of low-income, minority children receiving public health insurance. Because we did not assess behavioral supports within the home, school, or community, we are unsure whether or how other nutrition support programs may have played a role in the FVPP. Finally, the cross-sectional study design did not allow researchers to investigate the impact of the prescription program over time and assessments related to purchase and consumption of fruits and vegetables, as well as child-report of food security were not included. Still, this was an important preliminary study to examine associations between participation in a pediatric farmers’ market FVPP and farmers’ market shopping.

## 5. Conclusions

Children, particularly those living in poverty, often fail to meet dietary recommendations related to fruit and vegetable intake [7,8]. Given the positive association between participation in a pediatric FVPP and farmers’ market shopping, fruit and vegetable prescriptions written by primary care providers could have meaningful impacts on children’s dietary patterns. Future research will investigate whether, and to what degree, participation in FVPPs for pediatric patients is associated with long-term changes in food security, food access, and dietary patterns of children.

## Figures and Tables

**Table 1:** Characteristics of caregivers who completed a survey exploring participation in a fruit and vegetable prescription program for pediatric patients and farmers’ market shopping.

	Caregiver Characteristics	Frequency *n*	%
Gender	Female	146	93
	Male	11	7
Age	18–24	40	26
	25–34	58	37
	35–44	38	24
	45 and older	16	10
	No response	5	3
Education	Less than High School	21	13
	High School Graduate/GED	55	35
	Some College, No Degree	45	29
	Associate’s or Technical Degree	22	14
	Bachelor’s Degree or Higher	10	6
	No Response	4	3
City of Residence	Flint	113	74
	Outside Flint	44	26
	Child Characteristics	Frequency n	%
Gender	Female	135	52
	Male	125	48
Age	0–4	113	44
	5–11	88	34
	12–19	57	22
Race	Black/African-American	159	62
	White/Caucasian	66	26
	Other Responses	31	12

GED—General Equivalency Degree/Diploma.

**Table 2:** Differences in caregiver and child characteristics between fruit and vegetable prescription program participants versus non-participants.

Caregiver Characteristics		Participants *n* (%)	Non-Participants *n* (%)	*p*-Value for Difference by Program Participation
Gender	Female	84 (97.7%)	49 (87.5%)	0.015
	Male	2 (2.3%)	7 (12.5%)	
Age	18–24	23 (27.1%)	13 (24.1%)	0.311
	25–34	36 (42.4%)	17 (31.5%)	
	35–44	18 (21.2%)	19 (35.2%)	
	45 and older	8 (9.4%)	5 (9.3%)	
Education	Less than High School	10 (11.8%)	10 (18.2%)	0.420
	High School Graduate/GED	29 (34.1%)	17 (30.9%)	
	Some College, No Degree	30 (35.3%)	13 (23.6%)	
	Associate’s or Technical Degree	12 (14.1%)	10 (18.2%)	
	Bachelor’s Degree or Higher	4 (4.7%)	5 (9.1%)	
City of Residence	Flint	70 (81.4%)	20 (35.7%)	0.022
	Outside Flint	16 (18.6%)	36 (64.3%)	
**Child Characteristics**		**Participants**	**Non-Participants**	***p*-Value for Difference by Program Participation**
Gender	Female	77 (53.5%)	50 (48.5%)	0.445
	Male	67 (46.5%)	53 (51.5%)	
Age	0–4	70 (50.0%)	36 (36.0%)	0.088
	5–11	41 (29.3%)	40 (40.0%)	
	12–19	29 (20.7%)	24 (24.0%)	
Race	Black/African-American	100 (69.9%)	48 (47.5%)	<0.001
	White/Caucasian	25 (17.5%)	40 (39.6%)	
	Other Responses	18 (12.6%)	13 (12.9%)	

**Table 3:** Differences in household characteristics between fruit and vegetable prescription program participants and non-participants.

Household Characteristics	Total Sample	Participants	Non-Participants	*p*-Value for Difference by Program Participation
Total Responses	142	86	56	<0.001
WIC ^a^ Participant—*n* (%)	65 (45.8)	50 (58.1)	15 (26.8)	
Total Responses	140	85	55	0.990
SNAP ^b^ Participant—*n* (%)	89 (63.6)	54 (63.5)	35 (63.6)	
Total Responses	141	86	55	0.188
Double-Up Food Bucks ^c^ Participant—*n* (%)	34 (24.1)	24 (27.9)	10 (18.2)	
Total Responses	141	85	56	0.005
Farmers’ Market Shopping in Past Month—*n* (%)	58 (41.1)	43 (50.6)	15 (26.8)	
Total Responses	141	85	56	0.007
Farmers’ Market Shopping in Past Year—n (%)	94 (66.7)	64 (75.3)	30 (53.6)	
Total Responses	140	85	55	0.794
Low/Very Low Food Security—n (%)	63 (45.0)	39 (45.9)	24 (43.6)	
Total Responses	140	85	55	0.667
Food Security ^d^—mean ± SD	1.84 ± 1.98	1.89 ± 2.06	1.75 ± 1.89	
Total Responses	102	66	36	0.980
NFE ^e^—mean ± SD	257 ± 238	257 ± 240	258 ± 239	

^a^ WIC = Special Supplemental Nutrition Program for Women, Infants and Children; ^b^ SNAP = Supplemental Nutrition Assistance Program; ^c^ Duble-Up Food Bucks = Statewide fruit and vegetable incentive program that doubles the value of SNAP benefits spent at participating markets and grocery stores to purchase fresh fruits and vegetables; ^d^ US Household Food Security Module: Six Item Short Form, National Center for Health Statistics. Food security status assigned by raw score (0–1 = high/marginal food security; 2–4 = low food security; 5–6 = very low food security); ^e^ NFE = Neighborhood Food Environment score.
